# Asymmetric Interaction between *Aphis spiraecola* and *Toxoptera citricida* on Sweet Orange Induced by Pre-Infestation

**DOI:** 10.3390/insects11070414

**Published:** 2020-07-03

**Authors:** Jing Gao, Steve Arthurs, Runqian Mao

**Affiliations:** 1 Guangdong Key Laboratory of Animal Conservation and Resource Utilization, Guangdong Public Laboratory of Wild Animal Conservation and Utilization, Guangdong Institute of Applied Biological Resources, Guangdong Academy of Science, Guangzhou 510260, China; gaoj@giabr.gd.cn; 2 Biobee USA, Altamonte Springs, FL 32714, USA; stevenarthurs55@gmail.com

**Keywords:** aphid performance, feeding behavior, phytohormone-dependent defense, plant nutrition

## Abstract

Indirect interactions between herbivorous insects that share the same host have been focused on insects feeding on herbaceous plants, while few studies investigate similar interactions on woody plants. We investigated performance and feeding behavior of two citrus aphids, *Aphis spiraecola* Patch and *Toxoptera citricida* Kirkaldy, on sweet orange as affected by prior infestation of conspecifics and heterospecifics. Results showed that pre-infestation-induced interactions between *A. spiraecola* and *T. citricida* were asymmetric, with *A. spiraecola* gaining more fitness. In detail, pre-infestation by *A. spiraecola* decreased adult weight, enhanced survival rate and accelerated phloem sap acceptance of conspecifics. However, *A. spiraecola* pre-infestation did not affect performance or feeding behavior of *T. citricida*. In another infestation sequence, the pre-infestation of *T. citricida* did not affect conspecifics, but positively affected heterospecifics, indicated as a decreased pre-reproductive period, enhanced survival rate, adult weight, fecundity, and feeding efficiency, i.e., faster access and acceptance of phloem sap, and longer phloem sap ingestion duration. Furthermore, we found *A. spiraecola* pre-infestation enhanced amino acid concentration, amino acid to sugar ratio, activated salicylic acid and jasmonic acid marker gene expression, while *T. citricida* pre-infestation only depressed jasmonic acid marker gene expression. Changes in nutrient and phytohormone-dependent defense probably underlie the asymmetric effect.

## 1. Introduction

Infestation by herbivorous insects can indirectly affect the performance of subsequent herbivores positively, negatively, or neutrally, depending on the insect species, duration, and intensity of infestation [1,2]. In response to insect attack, plants show morphological and physiological changes [3]. These changes in plant quality may affect host selection, survival, fecundity, and population dynamics of any subsequent infested herbivores [4,5]. Therefore, these plant-mediated interactions may influence the population dynamics and community structure of herbivorous insects [6].

The performance of herbivorous insects can be affected by pre-infestation of both conspecific and heterospecific species. Many studies have measured plant-mediated inter-specific interactions between two herbivore species by transferring one insect species onto plants, and then measuring the survival, growth or insect number of the subsequently infested heterospecifics [7,8]. Similarly, the intra-specific interaction was detected by transferring insects onto plants and then measuring performance of subsequently infested conspecifics. The plant-mediated intra-specific effect may oppose or synergize that of the inter-specific effect. For example, conspecific pre-infestation increased the number of nymphs produced by *Myzus persicae* [9], while *Bemisia tabaci* pre-infestation reduced *M. persicae* fecundity [1]. Nevertheless, few studies have simultaneously investigated both intra- and inter-specific effects mediated by the same host plant [10]. Furthermore, while plant-mediated interactions between herbivorous insects have been widely reported for herbaceous plants, similar studies on woody plants have been largely overlooked. Moreover, in the latter case, most studies have focused on galling insects [11,12].

During the process of infestation, herbivorous insects induce multiple plant defense responses [13]. Phytohormone-dependent signaling, such as salicylic acid (SA) and jasmonic acid (JA), is important for plants to resist herbivore feeding [14]. While chewing insects mainly induce a JA-dependent defense, most sap-sucking insects such as aphids typically induce an SA-dependent defense [15]. These phytohormone-dependent defenses could affect the development and fecundity of subsequent infested insects. For example, the pre-infestation of *B. tabaci* induced an accumulation of SA, which prolonged developmental time and reduced fecundity of conspecifics [16]. An *Acyrthosiphon pisum* pre-infestation repressed JA synthesis, which decreased pre-reproductive period and increased fecundity of conspecifics [17]. In addition to plant-induced defense, nutrient change induced by herbivores may also influence the performance of subsequent infested herbivores. As nitrogen is often a limiting factor for herbivorous insects, the content of nitrogenous nutrition is often positively correlated with insect performance [18,19]. For example, *M. persicae* performed better on brassica plants that were already infested by conspecifics, due to a more favorable amino acid to sugar ratio [9].

Citrus is a globally important fruit tree, with exposure to a wide variety of insect pests [20]. Aphids are common pests that feed on citrus flush shoots and young leaves, impacting yields through decreased shoot growth, virus transmission, and facilitating fungus development by secreting honeydew [21,22]. Among them, *Toxoptera citricida* Kirkaldy and *Aphis spiraecola* Patch are common species and cause major damage [23]. *Toxoptera citricida* is oligophagous, feeding within the Rutaceae and is an efficient vector of Citrus tristeza virus [24]. By contrast *A. spiraecola* is more polyphagous, feeding on *Pyrus*, *Prunus*, *Malus*, and *Citrus* spp. [25]. *Aphis spiraecola* can transmit Citrus yellow vein clearing virus and Citrus tristeza virus [26,27].

In spite of the co-occurrence, the influence of plant-mediated intra- and inter-specific interactions between *A. spiraecola* and *T. citricida* has not been reported. In this paper, we quantified (1) life history parameters, including survival rate, adult weight, pre-reproductive period and fecundity of aphid feeding on plants pre-infested by conspecific and heterospecific individuals, (2) the feeding behavior of aphids tested by electrical penetration graph (EPG) technique as affected by conspecific and heterospecific pre-infestation, (3) total amino acid and soluble sucrose concentration and SA/JA marker genes expression in pre-infested citrus plants.

## 2. Materials and Methods

### 2.1. Experimental Plants and Aphid Culture

The sweet orange *Citrus sinensis* (L.) Osbeck were about 18 months old and were grown in 8 L pots. Plants were watered twice a week and fertilized with a 20–10–20 fertilizer solution (Haidesi fertilizer company, Weifang, Shandong, China) weekly. Plants were maintained in a growth chamber under 16:8 h photoperiod (day:night), 25 °C temperature, and 60–80% relative humidity. Two weeks before the experiment, plants were pruned to promote flushing.

Both *A. spiraecola* and *T. citricida* were obtained in 2018 from citrus orchards at Guangzhou, China. Wingless parthenogenetic females of two aphid species were separately maintained on different sweet orange plants in the growth chamber. Plants were covered with nylon mesh bags to prevent aphid dispersion.

### 2.2. Aphid Pre-Infestation Procedure

When shoots were approximately 2 cm long, 10 *A. spiraecola* or *T. citricida* fourth instar nymphs were transferred to a single new shoot and restricted by nylon mesh bags. After 48 h, the aphids were removed using a soft paint bush. Control shoots without aphids were similarly caged with nylon mesh bags for 48 h. Thus, there are three treatments (*A. spiraecola* pre-infestation, *T. citricida* pre-infestation, and control) in this study. The treated shoots were then infested with *A. spiraecola* or *T. citricida* to investigate plant-mediated indirect intra- and inter-specific effects. The number of treatments, aphid performance bioassay, and aphid feeding behavior analysis were described in the following sections.

Additional pre-infested and control shoots (five shoots per treatment) were excised, frozen in liquid nitrogen and stored at −80 °C for later analysis of amino acid concentration, sugar concentration, and gene expression.

### 2.3. Aphid Performance Bioassay

Several life history parameters, including survival rate, pre-reproductive period, fecundity, and adult weight were evaluated to determine the effects of pre-infestation on performance of subsequent aphids on the pre-infested and control shoots. To determine survival, seven newly emerged nymphs were introduced to each shoot and survival was recorded daily until the last nymph molting (six days). Survival rate (%) was calculated as the number of survived aphids divided by the number of total introduced aphids. After survival determination, the number of aphids on each shoot was adjusted to two to assess fecundity. Aphids were checked every 12 h to record the pre-reproductive period (days), and then every 24 h to record cumulative number of nymphs produced until adult aphids were 14 days old (early fecundity). Nymphs were removed daily to prevent populations increasing and facilitate counting. For the adult weight determination, six newly emerged nymphs were transferred to each pre-treated shoot. After 8 days, when aphids entered adult stage, their weights (mg) were measured individually with a microbalance (precision 0.1 mg). Aphid responses were measured from 15 shoots per treatment for each aphid species, and average data from one shoot was considered as a replicate.

### 2.4. Aphid Feeding Behavior

The electrical penetration graph (EPG) method can record the activity and locations of aphid stylets [28]. The feeding behaviors of *A. spiraecola* and *T. citricida* were studied according to the methods we previously used [29]. Apterous adults were starved for 8 h before experiments. Subsequently, a gold wire was attached to the dorsal side using conductive silver glue and connected to a copper extension wire inserted to an electro penetration graph (EPG) headstage amplifier. Another copper electrode was inserted into the soil. Individual aphids were placed on a shoot, and plants were placed in Faraday cages and subject to EPG monitoring over an 8 h period. The waveforms produced from EPG system included non-penetration (Np); pooled pathway phase activities (C), intracellular puncture (Pd), salivary secretion into sieve elements (E1), passive phloem ingestion (E2), and xylem absorption (G). Both con- and heterospecific pre-infestation treatments and control plants were included in the study, with 19–23 replicates per treatment.

The recorded EPGs were analyzed according to the waveform type and duration. A total of 12 parameters were analyzed. Six parameters are the total duration of the six described waveforms, another six parameters related to resistant against aphids are as follows [30]: (1) One parameter related to surface resistance is the time from onset of EPG recording to first probe (Pd); (2) Two parameters related to epidermis/mesophyll resistance and mesophyll/phloem resistance are the number of probes before first E1 and the minimum duration of C before first E1, respectively; (3) Two parameters reflecting the ease that aphids establish phloem access and acceptance are the time to first E1, and time to first sustained E2 from onset of EPG recording, respectively; (4) One parameter reflecting plant suitability is the average period of E2 (i.e., total time in E2 divided by the number of E2 waveforms per aphid).

### 2.5. Total Amino Acid and Soluble Sugar Concentration

Citrus leaves collected from five shoots per treatment (from 2.2) were assessed for total amino acid concentration. Leaves were used based on their similar relative amino acid concentration to that assessed from the phloem sap [31,32]. For each sample, 0.08 g leaf tissue was homogenized in a 0.72-mL phosphate buffer saline (0.01 mol/L, pH = 7) and centrifuged at 3500 rpm for 10 min. The supernatants were assessed using standard amino acid assay procedures of the total amino acid assay kit (Nanjing Jiancheng Bioengineering, Nanjing, China). Soluble sugar concentration was measured by the anthrone method using the plant soluble sugar content test kit from Nanjing Jiancheng Bioengineering. Approximately 0.07 g leaf per sample was homogenized in 1.8 mL distilled water. The mixture was boiled in water for 10 min and centrifuged at 4000 rpm for 10 min. Supernatants were diluted by distilled water and then used to detect sugar concentration according to standard procedures. Citrus leaves were assessed from five shoots per treatment.

### 2.6. Gene Relative Expression Detection

Total leaf RNA was isolated by TRIzol reagent (Invitrogen, Carlsbad, CA, USA) and 1 μg of the RNA was used to synthesis cDNA using the FastQuant RT Kit with gDNase (Tiangen, Beijing, China). The transcript levels of four target genes were analyzed by fluorescent real-time quantitative PCR: Non-expressor of Pathogenesis-Related genes 1 (*CtNPR1*), a well-known SA receptor; Pathogenesis related protein 1 (*CtPR1*), a SA marker gene, which encodes the SA inducing PR protein; allene oxide synthase (*CtAOS*), a gene for JA biosynthesis; and cysteine proteinase inhibitor (*CtPI*), which works downstream of JA, and encodes the JA inducing proteinase inhibitor. PCR was performed in 20-μL reaction volumes containing 10.4-μL 2× SYBR Premix (Tiangen, Beijing, China), 7.6-μL water, 1-μL gene-specific primers and 1-μL cDNA template. Reactions were carried out on the Mx 3500P detection system (Stratagene, La Jolla, CA, USA). For each biological replicate (4 per treatment), three technical repeats were performed. The transcript changes of the target genes were compared to the reference gene glyceraldehyde-3-phosphate dehydrogenase-1 (*CtGAPC1*). Primers were designed from gene sequences in NCBI or from published sequences [33,34] (Table 1).

### 2.7. Statistical Analysis

SPSS 20 software (SPSS Inc., Chicago, IL, USA) was used for statistical analysis. Aphids that died or shoots that defoliated during experiment were excluded from analysis. Data for survival rate and cumulative fecundity were analyzed with repeated measures ANOVA. One way ANOVA with Tukey’s HSD test was utilized for comparisons among the three treatments (conspecific pre-infestation, heterospecific pre-infestation, and control) for each aphid species. Results were deemed significantly different at *p* < 0.05. Proportion data associated with aphid survival rate were arcsine square root transformed to meet assumptions of normality and homogeneity of variance before analysis. The original data were used for presentation.

## 3. Results

### 3.1. Effects on Life-History Parameters

**Figure 1:**
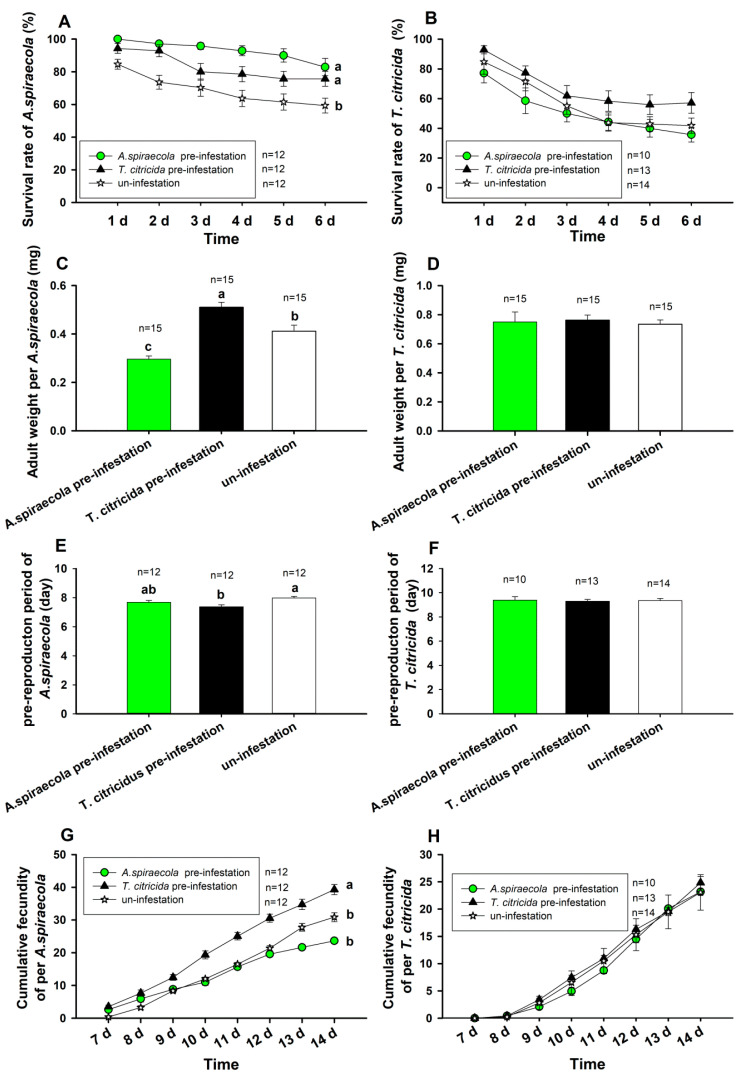
The life-history parameters of *A. spiraecola* and *T. citricida* feeding on sweet orange pre-infested by conspecific, heterospecific and without pre-infestation (control). The survival rate of *A. spiraecola* (**A**) and *T. citricida* (**B**), adult weight of *A. spiraecola* (**C**) and *T. citricida* (**D**), pre-reproductive period of *A. spiraecola* (**E**) and *T. citricida* (**F**), cumulative fecundity of *A. spiraecola* (**G**) and *T. citricida* (**H**) are represent the mean ± SEM. The numbers of replication are noted after treatment legends or above the bars. Different letters represent significant difference (Tukey’s HSD test, *p* < 0.05). Bars or lines without letters indicate there is no significant difference between treatments.

The differences were observed between the aphid species. The survival rate of *A. spiraecola* was higher on plants pre-infested with aphids of either species (versus controls) (Figure 1A) (*F*_2,33_ = 4.831, *p* < 0.001), although the same trend was not observed with *T. citricida* (Figure 1B) (*F*_2,34_ = 1.255, *p* = 0.298). The adult weight of *A. spiraecola* was lower when reared on plants pre-infested with conspecifics (*F*_2,42_ = 45.891, *p* < 0.001), although the opposite trend was observed when plants were previously infested with *T. citricida* (Figure 1C). No differences in adult weight were measured when *T. citricida* was added to pre-infested plants (Figure 1D) (*F*_2,42_ = 0.069, *p* = 0.933). Compared with controls, the pre-reproductive period of *A. spiraecola* was lower on plants previously infested with *T. citricida* but not with conspecifics (Figure 1E) (*F*_2,33_ = 7.353, *p* = 0.002). *Toxoptera citricida* did not show difference in pre-reproductive period by pre-infestation (Figure 1F) (*F*_2,34_ = 0.038, *p* = 0.963). Finally, *A. spiraecola* exhibited increased reproductive output over 14 days on plants pre-infested with *T. citricida,* but not with conspecifics (Figure 1G) (*F*_2,33_ = 37.338, *p* < 0.001). No similar trend in reproductive output was measured when *T. citricida* was added to pre-infested plants (Figure 1H) (*F*_2,34_ = 1.223, *p* = 0.307).

### 3.2. Effects on Feeding Behavior via EPG Measurements

**Figure 2:**
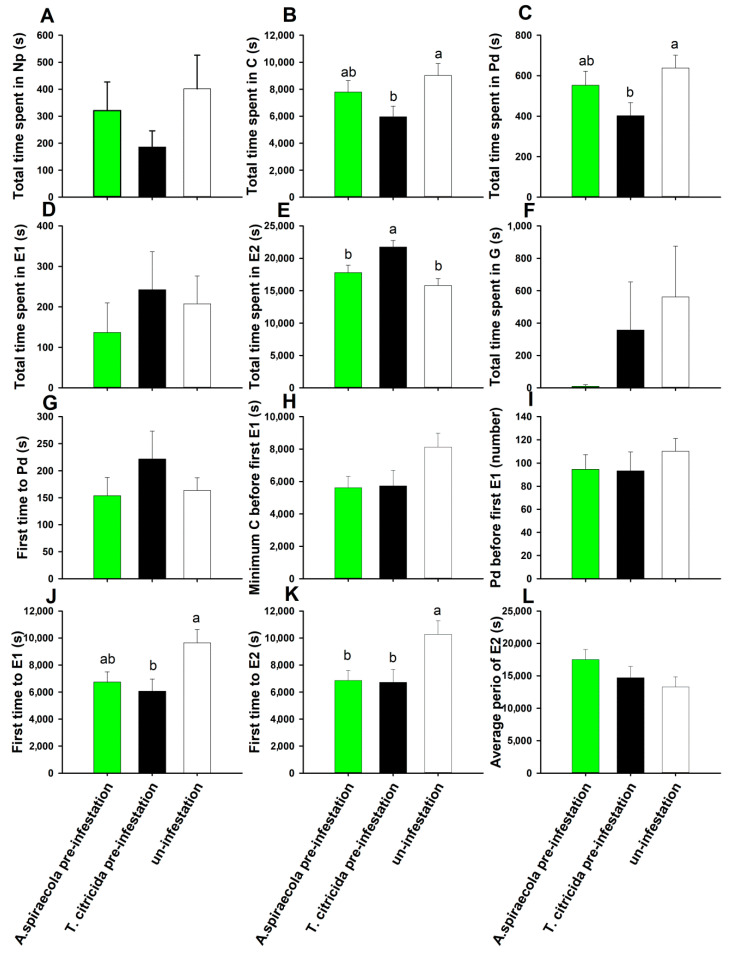
Effect of conspecfic and heterospecific pre-infestation on the feeding behavior of *A. spiraecola*. Values represent the mean ± SE (n = 21–22). Bars with different letters represent significant difference (Tukey’s HSD test, *p* < 0.05) in each waveform event. Bars without letters indicate there is no significant difference between treatments.

**Figure 3:**
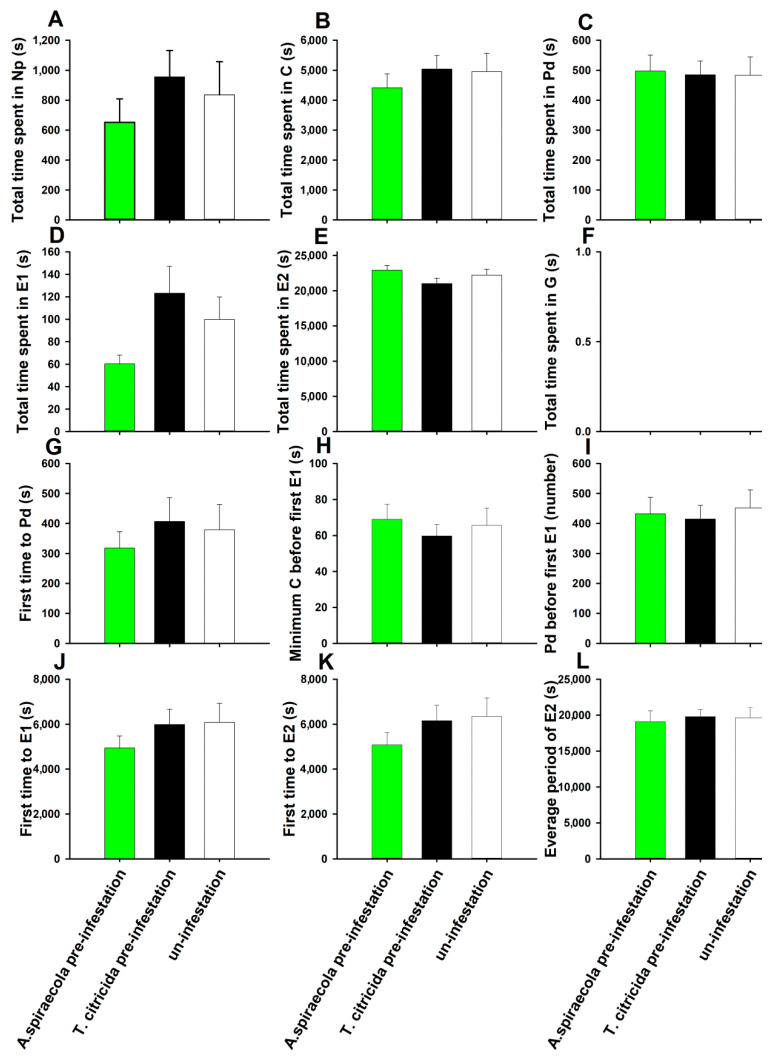
Effect of conspecfic and heterospecific pre-infestation on the feeding behavior of *T. citricida*. Values represent the mean ± SE (n = 19–23). Bars with different letters represent significant difference (Tukey’s HSD test, *p* < 0.05) in each waveform event. Bars without letters indicate there is no significant difference between treatments.

*Aphis spiraecola* feeding on plants pre-infested by conspecifics required less time to reach sustained phloem sap ingestion compared with controls (Figure 2K) (*F*_2,61_ = 5.921, *p* = 0.010), although other feeding parameters as measured by EPG were unaffected (Figure 2) (*p* > 0.05). More differences were observed when plants were pre-infested with *T. citricida* (heterospecific pre-infestation). In this case, *A. spiraecola* exhibited reduced time of pathway phase activity (Figure 2B) (*F*_2,61_ = 3.235, *p* = 0.046), intracellular probe (Figure 2C) (*F*_2,61_ = 3.911, *p* = 0.025), and reduced the duration before the first sieve element salivation (Figure 2J) (*F*_2,61_ = 4.612, *p* = 0.014) and before the first sustained phloem sap ingestion (Figure 2K). The duration of phloem sap ingestion by *A. spiraecola* was also increased on plants pre-infested with heterospecifics (Figure 2E) (*F*_2,61_ = 7.761, *p* = 0.001). For *T. citricida,* feeding on plants pre-infested by either aphid species did not affect the twelve feeding activity parameters (Figure 3) (*p* > 0.05). Moreover, unlike *A. spiraecolai*, *T. citricida* had no xylem absorption activity.

### 3.3. Amino Acid and Soluble Sugar Concentration

**Figure 4:**
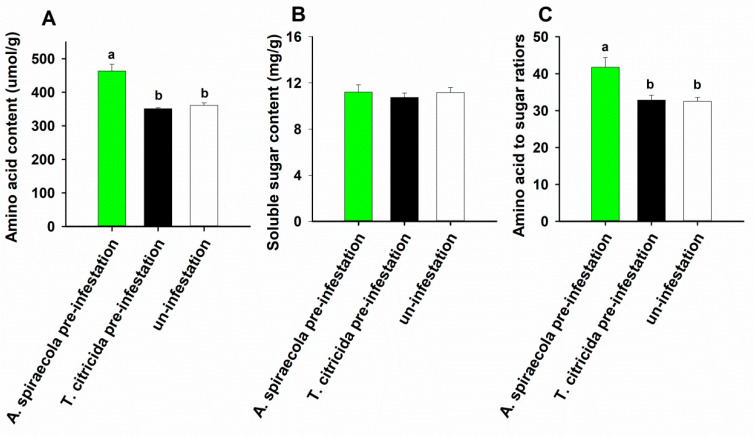
Amino acid concentration (**A**), soluble sugar concentration (**B**), and amino acid to sugar ratios (**C**) in sweet orange leaves pre-infested by *A. spiraecola*, *T. citricida*, or without aphid pre-infestation. Values represent the mean ± SE (Tukey’s HSD test, n = 5). Bars with different letters represent significant difference (*p* < 0.05). Bars without letters indicate there is no significant difference between treatments.

Infesting sweet orange plants with *A. spiraecola* enhanced total amino acid concentration (Figure 4A) (*F*_2,12_ = 24.199, *p* < 0.010) and amino acid to sugar ratio (Figure 4C) (*F*_2,12_ = 8.306, *p* = 0.005) but did not affect soluble sugar concentration in sweet orange (Figure 4B) (*F*_2,12_ = 0.245, *p* = 0.787). Similarly, no significant difference was observed when plants were infested with *T. citricida* compared with control (*p* > 0.05).

### 3.4. SA and JA Marker Gene Expression in Sweet Orange

**Figure 5:**
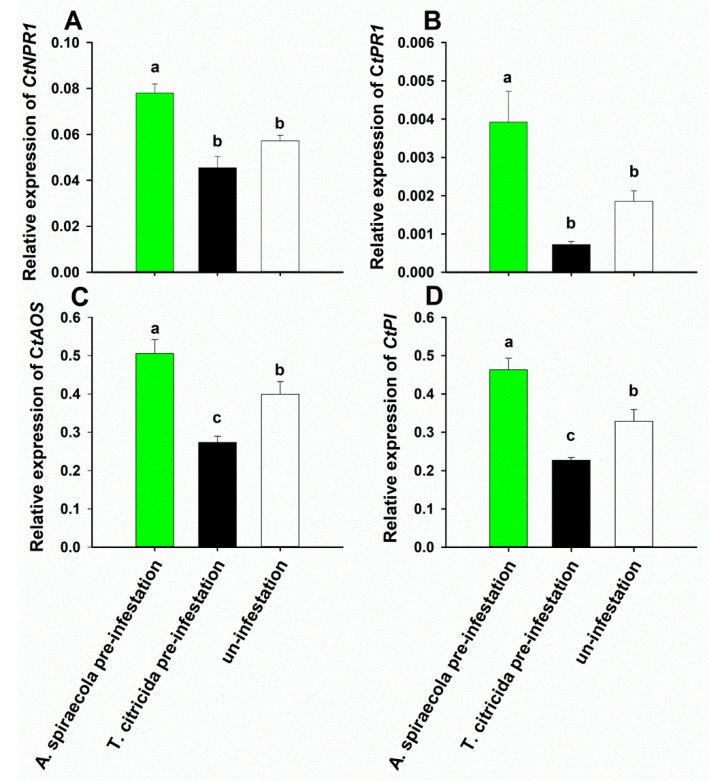
Relative expression of salicylic acid (**A**,**B**) and jasmonic acid (**C**,**D**) marker genes in *A. spiraecola* pre-infested, *T. citricida* pre-infested, or un-infested sweet orange. Gene expression levels were evaluated relative to *CtGAPC*. Values represent the mean ± SE (n = 4). Bars with different letters represent significant difference (Tukey’s HSD test, *p* < 0.05).

Infesting sweet orange plants with *A. spiraecola* enhanced the expression of SA marker genes *CtNPR1* (*F*_2,33_ = 24.423, *p* < 0.001) and *CtPR1* (*F*_2,33_ = 12.622, *p* < 0.001), the JA marker genes *CtAOS* (*F*_2,33_ = 18.752, *p* < 0.001) and *CtPI* (*F*_2,33_ = 27.174, *p* < 0.001) (Figure 5) compared with *T. citricida* infestation or control. By contrast, *T. citricida* infestation depressed JA, but not SA marker gene expression, when compared with controls.

## 4. Discussion

Here, we found an asymmetrical interaction on the performance and feeding behavior of two citrus aphids. We observed that *A. spiraecola* gained enhanced survival rate from conspecific pre-infestation, and gained fitness benefits in survival rate, adult weight, and fecundity from heterospecific pre-infestation. However, *T. citricida* was not affected by either conspecific or heterospecific pre-infestation. Pre-infestation-induced interaction favors *A. spiraecola* as a superior competitor, and may influence the population and community of citrus aphid species.

The reciprocal effects of different herbivore species that share the same host plant have gained much attention. We found a positive effect on *A. spiraecola* and neutral effect on *T. citricida* induced by heterospecific pre-infestation. This is different to the most reported detrimental effect on other species, such as whitefly pre-infestation negatively affected performance of *M. persicae* [35], leaf miners [36,37], and *Pieris rapae* [38], which may help whitefly become a strong competitor. Here, *A. spiraecola* gains fitness benefit in the interspecific interaction through promoting performance of itself rather than inhibiting other species. In addition to the performance change, feeding behavior detected by EPG technique allows quantification of aphid response, and may help explain how aphid are affected by pre-infestation [39]. *Aphis spiraecola* on heterospecific pre-infested plant spent less time on penetration or pathway phase, spent more time on phloem sap ingestion, and gained quicker access and acceptance of phloem sieve elements. These feeding behavior parameters indicate heterospecific pre-infestation enhanced plant susceptibility to *A. spiraecola* [40,41], which was consistent with enhanced performance. On the other hand, in line with the performance, feeding behavior of *T. citricida* was also not affected by *A. spiraecola* pre-infestation. *Toxoptera citricida* pre-infestation showed a positive effect on *A. spiraecola*; thus, the management of *T. citricida* may reduce the fitness of *A. spiraecola*, indicating additional secondary benefits of controlling *T. citricida*.

Sap-sucking insects like aphids often aggregate in the feeding site, thus conspecific pre-infestation usually occurs to affect insect performance [42]. Con-specific pre-infestation enhanced the survival rate, decreased adult weight, and decreased the time spent before phloem sap ingestion for *A. spiraecola* compared with control. It has been shown that *A. spiraecola* feeding caused leaf curling, which would reduce the space provided for aphids. In addition, the enhanced survival rate caused by conspecific pre-infestation increased the number of aphids. These factors would enhance the density of *A. spiraecola*, a factor important in producing the winged form of aphids [43,44]. The winged form would help it disperse to more new shoots or plants. Therefore, conspecific pre-infestation is thought to favor *A. spiraecola* fitness. In contrast, *T. citricida* pre-infestation did not affect performance or feeding behavior of con-specifics. When considering both the intra- and inter-specific effects, for *A. spiraecola*, the conspecific pre-infestation effect is less strong than that of heterospecific (only the enhanced survival rate vs enhanced performance of all the tested life history parameters), while for *T. citricida*, the conspecific and heterospecific effect did not differ (both are unchanged). Thus, the pre-infestation may intensify inter- more than intra-specific competition between citrus aphids. Besides, the conspecific and heterospecific pre-infestation induced a positive synergistic effect on *A. spiraecola*, which may help explain why it has become a dominant species in the citrus groves in America [23].

Herbivore-induced changes in host plant morphology, nutrition, defense, or some combination of these changes mediated an alteration of the performance of subsequent infested insects [45,46,47]. In our study, *T. citricida* pre-infestation did not affect SA signaling marker genes expression, but depressed those involved in JA signaling. As the JA-dependent defense is considered effective in conferring resistance against phloem-sucking insects [48], the depressed defense may benefit subsequently infested aphids. Concurrently, we observed that *A. spiraecola* feeding on heterospecific infested plants had better performance and enhanced feeding efficiency. Particularly, the less time before reaching phloem sap and passive phloem ingestion also indicate the decreased mesophyll/phloem resistance it encountered [30]. Interestingly, plants pre-infested with *A. spiraecola* showed increased SA and JA signaling gene expression, but did not affect the performance of subsequent infested *T. citricida*, indicating that other factors such as nutrition change may involve. Amino acid concentration and amino acid to sugar ratio are considered as an index of host plant nutrient quality for aphids [18]. Positive correlations between growth, reproduction and plant amino acid concentration were established in *Rhopalosiphum insertum* and *M. persicae* [9,49]. The infestation of *A. spiraecola* enhanced the amino acid concentration and amino acid to sugar ratio. We hypothesis that the beneficial effect of enhanced nutrition counteract the detrimental effect of induced defense, which results in unchanged performance of *T. citricida* by heterospecific pre-infestation. For the conspecific interaction, even feeding on plant with enhanced phytohormone-dependent defense, *A. spiraecola* feeding behavior reflects the fact that it encountered a decreased mesophyll/phloem resistance. It is possible that *A. spiraecola* can overcome the induced defense, and the decreased adult weight reflects energy cost of detoxification [50]. Therefore, both pre-infestation-induced changes in defenses and nutrition were involved in the indirect interaction between citrus aphids. However, although *T. citricida* repressed JA defense, the performance and feeding behavior of subsequent infested conspecific is not affected, may be *T. citricida* is less likely to be affected by plant defense through the long-term co-evolution with host plant. The quality of the plant after insect feeding on a pre-infested plant will help fully explain pre-infestation effect on conspecifics and heterospecifics.

## 5. Conclusions

To our knowledge, this is the first study to investigate plant-mediated effects on performance and feeding behavior of different citrus aphids. *A. spiraecola* and *T. citricida* show asymmetric interaction induced by pre-infestation. Particularly, *T. citricida* pre-infestation caused *A. spiraecola* to gain more fitness. Furthermore, pre-infestation-induced changes in phytohormone-dependent defense and nutritional quality probably underlie the asymmetric interaction. Moreover, the two citrus aphids manipulate host plant physiology in distinct ways, which may relate to the different adaptive strategy between oligophagous and polyphagous insects through the long term co-evolution with host plant [51].

## Figures and Tables

**Table 1:** Primers used in present study.

Gene Function	Gene	Primer Sequence (5’–3’)
Reference gene	*CtGAPC1*	F: ACTCCAGAGGGATGATGTGG
		R: ATGGGATCTCCTCTGGGTTC
Salicylic acid	*CtNPR1*	F: TGATAAGACCTTGCCACAACAC
signaling		R: ACCGCAGGATTCAGATCTATGT
	*CtPR1*	F: ACTGCAATCTTGTGCATTCG
		R: TTCACCCACAGTTTCACAGC
Jasmonic acid	*CtAOS*	F: GTTTCAGCTCGCTCCGTTAC
signaling		R: GAGGTTGTGACACGCTTCCT
	*CtPI*	F: AATCTTCTCATCGCTTTATC
		R: TGCTTCGCACTTACAACT
